# Dual-tracer PET/CT imaging to determine tumor heterogeneity in a patient with metastatic ACTH-secreting neuroendocrine neoplasm: A case report and literature review

**DOI:** 10.3389/fendo.2022.958442

**Published:** 2022-09-05

**Authors:** Daria Ryzhkova, Lubov Mitrofanova, Uliana Tsoy, Elena Grineva, Evgeny Schlyakhto

**Affiliations:** ^1^ Nuclear Medicine Department, Almazov National Medical Research Center, Saint Petersburg, Russia; ^2^ Department of Pathomorphology, Almazov National Medical Research Center, Saint Petersburg, Russia; ^3^ Department of Neuroendocrinological Tumors, Almazov National Medical Research Center, Saint Petersburg, Russia; ^4^ Institution of Endocrinology, Almazov National Medical Research Center, Saint Petersburg, Russia; ^5^ Almazov National Medical Research Center, Saint Petersburg, Russia

**Keywords:** PET/CT, [18F]-FDG, [68Ga]-DOTATATE, ectopic Cushing’s syndrome, ovarian NET

## Abstract

**Introduction:**

We present a case of a patient with disseminated ACTH-secreting neuroendocrine neoplasm with biologic heterogeneity between a primary tumor and metastases. The diagnosis was obtained and multidisciplinary management was conducted with a positron emission tomography/computed tomography (PET/CT) scan with Gallium-68 [68Ga]-labeled dodecanetetraacetic acid-tyrosine-3-octreotate ([68Ga]-DOTA-TATE) and Fluor-18 [18F]-fluorodeoxyglucose ([18F]-FDG).

**Case report:**

A PET/CT scan revealed a difference between [68Ga]-DOTA-TATE and [18F]-FDG uptake in primary tumor and several metastases. PET/CT showed high [18F]-FDG uptake and lack of [68Ga]-DOTA-TATE in the primary tumor, whereas both [68Ga]-DOTA-TATE and [18F]-FDG hyperaccumulation were identified in the majority of metastases. Despite positive [68Ga]-DOTA-TATE PET/CT, which is associated with high affinity with the somatostatin receptor 2 subtype, immunohistochemical examination revealed overexpression of the somatostatin receptor 5 subtype only. Perhaps, this explained the ineffectiveness of the treatment with “cold” somatostatin analogs.

**Conclusion:**

This case had an aggressive clinical course, despite cytoreductive surgical treatment and somatostatin analog therapy. PET/CT imaging with two tracers is a molecular tool that demonstrates a biologic heterogeneity between a primary tumor and metastases and yields additional information that may influence the choice of the patient management strategy.

## Introduction

Neuroendocrine tumors (NETs) are a type of neoplasms that arise from neuroendocrine cells, spread throughout the body, and exhibit symptoms related to the secretion of bioactive amines and peptides. The functional and phenotypical differences between a primary neuroendocrine tumor and metastases are usually explained by genetic changes in cancer cells leading to the loss of their differentiation. The tumor heterogeneity represents a significant challenge for the prognosis and choice of optimal treatment strategies for NETs. However, the multiregion sampling from each tumor of a single patient is not always possible due to technical and ethical reasons. Hybrid positron emission tomography and computer tomography (PET/CT) is a whole-body non-invasive method that allows assessing tumor heterogeneity through the use of a range of radiopharmaceuticals, each providing different and complementary information.

We report a case of a patient with ovarian disseminated adrenocorticotropic hormone (ACTH)-secreting tumor, where heterogeneity was explored with multiple tracer PET/CT imaging.

## Case presentation

A 53-year-old woman was referred to our Department of Endocrinology, with a 7-month history of muscular weakness, truncal obesity, hyperglycemia, skin darkening, shortness of breath, and cough. The patient had menopause at the age of 42 and arterial hypertension at the age of 50. The patient had taken hypotensive therapy with bisoprolol 10 mg per day, perindopril 10 mg per day, and indapamide 2.5 mg per day and had her blood pressure stabilized within 130/80–140/90 mmHg. Since the age of 51, she has begun to notice an increase in blood pressure to 180/110–190/120 mmHg.

Laboratory examination revealed hyperglycemia (10.5 mmol/L) and a slight increase of morning serum cortisol concentration of 557.3 nmol/L, collected at 8:00 a.m. (normal, 101.2–535.7 nmol/L), which was not suppressed by 1 mg of dexamethasone (477.9 nmol/L). Serum ACTH concentration was 248.9 pg/ml (normal, <63 pg/ml). Basal, 24-h urine cortisol level was 1,154.9 nmol/24 h (normal, 11.80–485.60 nmol/24 h). Laboratory tests showed chromogranin A concentration of 595.71 µg/ml (normal, <100 µg/ml).

Magnetic resonance imaging of the chiasmal–sellar region did not identify pituitary adenoma. Bilateral simultaneous sampling of the inferior petrosal sinuses excluded Cushing disease as the cause of the high level of ACTH.

An ectopic ACTH syndrome was diagnosed, and the patient was referred for chest and abdomen CT, colonoscopy, and bronchoscopy to determine the location of the ACTH-secreting neuroendocrine tumor. Computed tomography showed mediastinal lymphadenopathy and multiple osteoblastic lesions of the ribs and vertebrae, in which metastases were suspected. Colonoscopy and bronchoscopy did not reveal any signs of NET.

**Figure 1:**
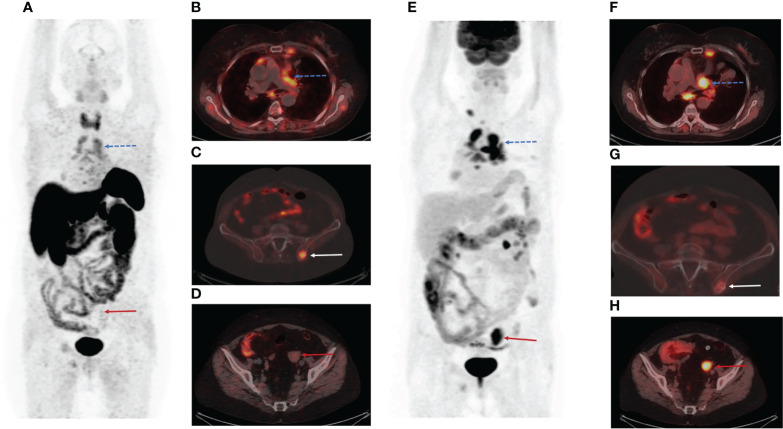
Positron emission tomography/computed tomography (PET/CT) images with Gallium-68 [68Ga]-labeled dodecanetetraacetic acid-tyrosine-3-octreotate ([68Ga]-DOTA-TATE) and Fluor-18 [18F]-fluorodeoxyglucose ([18F]-FDG). **(A)** The maximum intensity projection (MIP) images summarize the physiological and pathological distribution of [68Ga]-DOTA-TATE in the body. **(B)** PET/CT scan showed multiple mediastinal lymphatic nodes (blue dotted arrow) and widespread bone metastases with significant uptake of [68Ga]-DOTA-TATE. **(C)** [68Ga]-DOTA-TATE-positive bone metastasis in the left iliac wing (white arrow). **(D)** [68Ga]-DOTA-TATE-negative solid mass in the left ovary (red arrow). **(E)** The MIP images summarize the physiological and pathological distribution of [18F]-FDG in the body. **(F)** Multiple [68Ga]-DOTA-TATE-positive metastases in mediastinal lymphatic nodes (blue dotted arrow). **(G)** The axial PET/CT image demonstrated low [18F]-FDG uptake in the bone metastasis in the left iliac wing (white arrow). **(H)** The axial slice of [18F]-FDG PET/CT showed high [18F]-FDG uptake in the solid mass in the left ovary (red arrow).

The patient underwent PET/CT with [68Ga]-DOTA-TATE. [68Ga]-DOTA-TATE was synthesized in compliance with the European Pharmacopoeia (Gallium[68Ga] Edotreotide injection 01/2013: 2482 corrected 8.6). PET/CT scan was performed in 60 minutes after intravenous administration of 150 MBq of radiopharmaceutical. PET/CT scan showed multiple mediastinal lymphatic nodes, and widespread bone metastases with significant uptake of [68Ga]-DOTA-TATE (**Figures 1A–C**). Moreover, PET/CT revealed [68Ga]-DOTA-TATE negative solid mass in the left ovary (size 34x30x47 mm) (**Figure 1D**), peritoneal implants, and enlarged right cervical lymphatic node.

We assumed that the patient had poor differentiated neuroendocrine tumor in the left ovary. The patient was referred for PET/CT with Fluor-18 [18F]-fluorodeoxyglucose ([18F]-FDG), which was performed three days after the first PET/CT. PET/CT [18F]-FDG showed high uptake of the radiopharmaceutical in a solid mass of the left ovary (SUV max= 6,49) (**Figures 1E, H**), peritoneal implants (SUV max= 1,54), right cervical lymphatic node (SUV max= 3,91), and several mediastinal lymphatic nodes (SUV max= 3,67) (**Figures 1E, F**). However, we observed accumulation of [18F]-FDG in only a few bone metastases (**Figure 1G**).

Surgical excision of [18F-FDG]-avid right cervical lymphatic node and endoscopic ultrasonography-guided biopsy of mediastinal lymphatic node with high uptake of [68Ga]-DOTA-TATE were performed, and morphological examination revealed the neuroendocrine origin for both. The cervical lymphatic node was 2 × 1.2 × 1 cm and gray on the section. Histological examination showed that 90% of it was replaced by tumor metastasis, consisting of nests and tubules of structures of cells with a distinct cytoplasm and nuclei with a “salt–pepper” effect. Immunohistochemistry showed the expression of CD56, сhromogranin A, synaptophysin, and SSTR 5 in tumor cells. The Ki-67 index was 10%–15% and 10% of the tumor cells expressed ACTH. SSTR 2 expression was not detected. A biopsy of the mediastinal lymphatic node guided by endoscopic ultrasonography was performed through the wall of the esophagus. Uniform rounded small cells expressing synaptophysin were found in the aspirate and Ki-67 was 8%–9%. The diagnosis of neuroendocrine tumor metastases in the lymphatic nodes was made.

A genetic study using panel of genes associated with the development of genetically determined neuroendocrine tumors (*AIP, APC, CDKN1B, CDKN1C, EPAS1, GNAS, HABP2, HRAS, MAP3K1, MAX, MC2R, MEN1, NF1, NRAS, NTRK1, PRKACA, PRKAR1A, RET, SDHA, SDHAF2, SDHB, SDHC, SDHD, TMEM127, TP53, TSC1, TSC2, VHL, ZNRF3*>) was performed with a new generation sequencing method. It did not reveal any pathogenic variants or variants of uncertain significance.

The patient underwent bilateral adrenalectomy due to the severity of manifestations of hypercortisolism and for the prevention of complications. An exploratory laparotomy was performed a month later. Intraoperatively, a large left ovarian tumor measuring 4 × 4.5 × 4 cm was found. The adjacent peritoneal surface showed several metastatic nodules measuring up to 2.5 cm in diameter. The tumor together with the right ovary, fallopian tubes, and the adjacent peritoneal nodules was removed.

**Figure 2:**
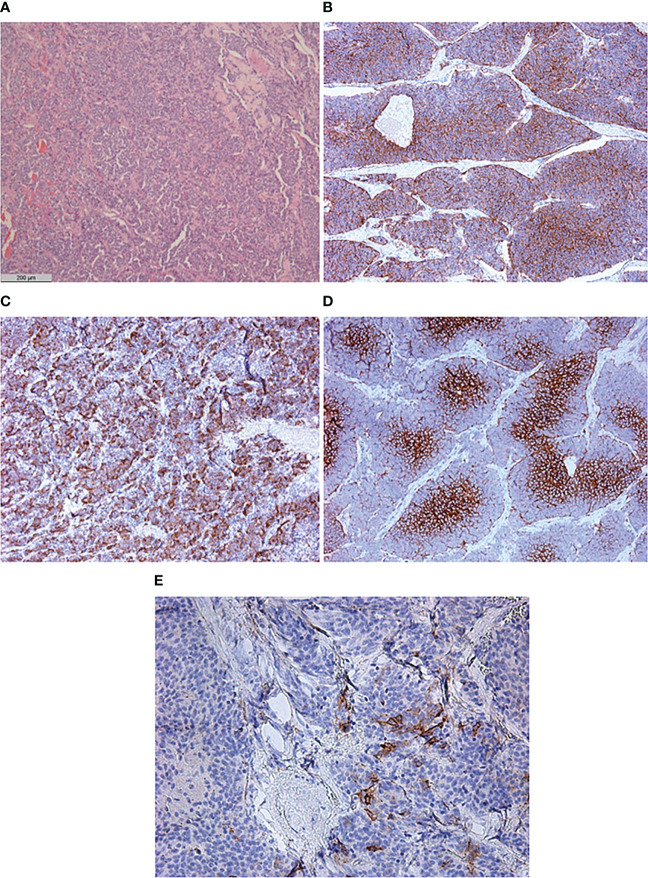
Histological and immunohistochemical examination of primary neuroendocrine tumor of the left ovary. **(A)** Hematoxylin and eosin staining ×50. **(B)** CD56 ×50. **(C)** ACTH ×50. **(D)** GLUT-1 ×100. **(E)** SSTR 5 subtype ×200.

Histopathological examination of the excised left ovary demonstrated subtotal replacement of healthy tissue by a solid yellowish-gray tumor with hemorrhage and fields of solid growth of large cells with polymorphic nuclei with “salt and pepper” chromatin. Tumor invasion into the vessels, ovarian capsule, and left fallopian tube was noted. Immunohistochemistry revealed positivity for chromogranin A, synaptophysin, neuron-specific enolase (NSE), and ACTH, and the Ki-67 index was 15%. Based on these findings, a diagnosis of ovarian non-small cell neuroendocrine tumor was made. Interestingly, only 5% of the tumor cells overexpressed the somatostatin receptor 5 subtype, and none of them expressed the somatostatin receptor 2 subtype. GLUT-1 overexpression was seen in 85% of the tumor cells (**Figure 2**). The right ovary and fallopian tubes were free of tumor growth. A diagnosis of a primary neuroendocrine tumor of the left ovary with ACTH production (grade 2) was made.

Serum ACTH concentration decreased from 248.9 to 167 pg/ml after surgery. Treatment with the somatostatin analog lanreotide autogel 120 mg every 28 days was prescribed due to disseminated disease and [68Ga]-DOTA-TATE-positive multiple metastases in the lymphatic nodes and bones.

**Figure 3:**
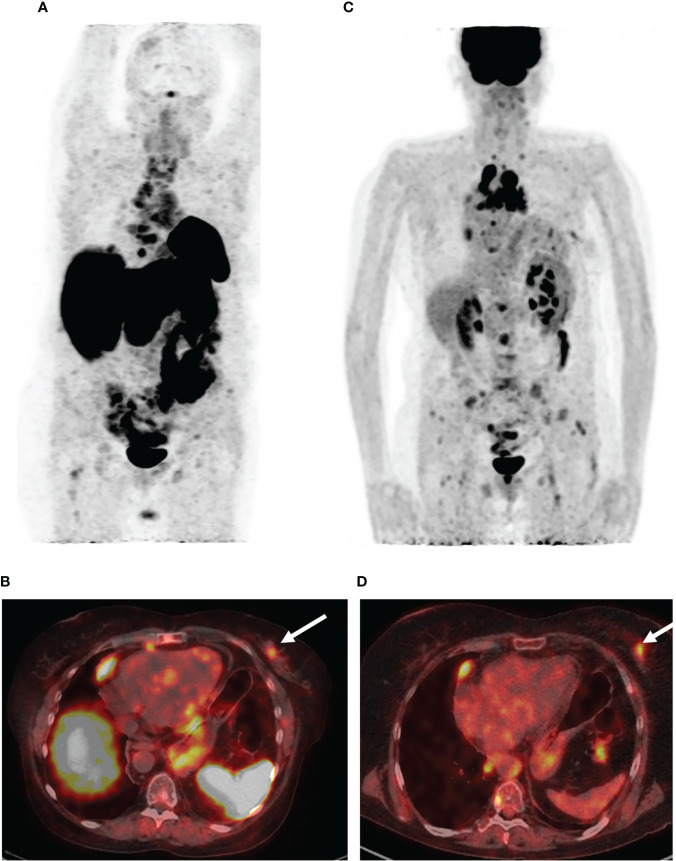
PET/CT images with [68Ga]-DOTA-TATE and [18F]-FDG. **(A)** The maximum intensity projection (MIP) images summarize the physiological and pathological distribution of [68Ga]-DOTA-TATE in the body. PET/CT scan showed an increase in the size of the previously detected metastases and the appearance of a new mediastinal lymph node and bone metastases. **(B)** [68Ga]-DOTA-TATE. PET/CT revealed an avid metastatic lesion in the upper inner quadrant of the left breast (white arrow). **(C)** The MIP images of [18F]-FDG PET showed intense tracer uptake in the same area as the [68Ga]-DOTA-TATE PET/CT scan. **(D)** [18F]-FDG-positive metastatic lesion in the left breast (white arrow).

**Figure 4:**
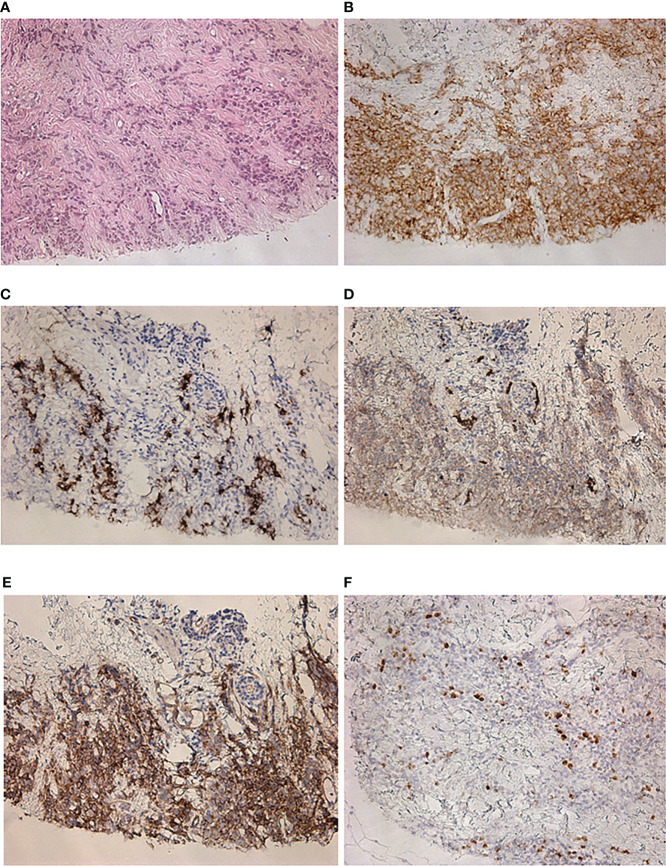
Histological and immunohistochemical examination of a metastatic lesion in the left breast. **(A)** Hematoxylin and eosin staining ×200. **(B)** Synaptophysin ×200. **(C)** ACTH ×200. **(D)** GLUT-1 ×200. **(E)** SSTR 5 subtype ×200. **(F)** Ki-67 ×200.

Five months after the surgical treatment, the patient underwent [68Ga]-DOTA-TATE PET/CT scan to assess the efficacy of the pharmacological treatment. This showed an increase in the size of the previously detected metastases and the appearance of a new mediastinal lymph node and bone metastases, while the radiopharmaceutical uptake did not have a significantly effect (**Figure 3A**). A month before the first admission to the endocrinology department, mammography was performed, which showed only fibrocystic breast disease with a BI-RADS score of 2. However, the follow-up [68Ga]-DOTATATE PET/CT scan now showed an avid metastatic lesion in the upper inner quadrant of the left breast (**Figure 3B**) that was not visualized during the previous PET/CT scans. An [18F]-FDG PET/CT scan showed intense tracer uptake in the same area as the [68Ga]-DOTA-TATE PET/CT scan, indicating left breast metastasis of the ovarian NET (**Figures 3C, D**). Ultrasound imaging identified a 2.2 × 1.1-cm hypoechoic mass with irregular contours and vascularization as shown by color Doppler sonography. An ultrasound-guided 18-G needle core biopsy was performed and the histological evaluation showed a neuroendocrine tumor of nests and tubules with “salt and pepper” nuclei. Immunocytochemistry showed the expression of chromogranin A, synaptophysin, NSE, and ACTH, and the Ki-67 index was 15% (**Figure 4**). The majority of the tumor cells expressed the somatostatin receptor 5 subtype (**Figure 4E**). These findings were similar to the immunocytochemistry of the previous metastases and the primary neuroendocrine tumor of the ovary.

Based on the PET/CT results, the treatment course was modified to systemic chemotherapy with standard doses of carboplatin and рaclitaxel in addition to the somatostatin analog.

## Discussion

ACTH-producing tumors arise from various organs. Cushing’s syndrome is often associated with small cell lung cancer and pancreas and thymus NETs. The ACTH-producing NETs of other localizations, including the ovaries, are extremely rare (1–6). Ovarian NETs account for only 0.1% of all ovarian neoplasms and less than 5% of all neuroendocrine tumors (7). Neuroendocrine tumors of the ovary may be primary or metastatic, primary ovarian NETs are often unilateral, and metastatic tumors are often bilateral and may be multinodular in each ovary (7, 8). Primary ovarian NETs may develop in pure form or arise within a cystic teratoma or dermoid tumor (7, 9, 10). Ki et al. (11) proposed several hypotheses of the histogenesis of ovarian neuroendocrine tumors. The first hypothesis assumed that neuroendocrine cells of the female genital tract serve as the origin of neuroendocrine tumors of the ovary. The other hypotheses suggested that the development of a neuroendocrine tumor may be due to the activation of genes promoting neuroendocrine differentiation in ovary, primitive endocrine, or non-neuroendocrine cells (12, 13).

The treatment strategy for ovarian NETs should take into account the stage, histological type, patient age, and fertility expectation (14). Primary carcinoid tumors typically behave in a benign fashion, and the optimal treatment is surgical resection. Systemic chemotherapy, therapy with radionuclides, and palliative surgery are effective methods for advanced neuroendocrine tumors. However, according to research, the biological and histological characteristics of NETs are also critical for planning further treatment (15).

The main strength of nuclear medicine is in the different radiopharmaceuticals allowing to explore the organs and systems of the body. The radiopharmaceuticals are important labeled biomarkers for personalized medicine and individualized treatment. Tumor heterogeneity contributes to errors in the choice of the treatment strategy, especially in metastatic diseases, because the expression of targets for therapy in the primary tumor does not guarantee their expression in metastases. PET/CT imaging overcomes these limitations by detecting heterogeneity in the primary tumor and metastases, as well as identifying target expression throughout the whole body, which is essential for clinical decision-making.

Nowadays, there are multiple radiotracers that target somatostatin receptors. [68Ga]-DOTA-TATE is a radiopharmaceutical that has affinity to somatostatin receptors, which are expressed by most well-differentiated neuroendocrine tumors and can be used as targets for radionuclide imaging and therapy (16). [18F]-FDG PET/CT is traditionally performed for poorly differentiated tumors. The research by Deroose et al. (17) emphasized the usefulness of [18F]-FDG PET/CT to complement somatostatin receptor imaging in well-differentiated grade G2 neuroendocrine tumors. They reported that the mismatch between somatostatin receptor imaging and [18F]-FDG PET/CT accurately represented tumor differentiation, and the high uptake of [18F]-FDG is a valuable marker of high-grade neuroendocrine tumors. In the study reported by Sehouli et al. (18), a potential diagnostic algorithm was proposed for patients with ovarian neuroendocrine neoplasms based on PET/CT data with different radiopharmaceuticals. This algorithm included somatostatin receptor imaging by either octreotide scintigraphy or [68Ga]-DOTATOC PET/CT. Chan et al. (19) proposed the strategy of initial identification of the lesion that had been more FDG-avid, than [68Ga]-DOTA-peptides, and this lesion had been likely to represent the most aggressive phenotype in patients with metastatic neuroendocrine tumor. Moreover, the authors designed the NETPET scoring system, where a grade P1 indicated somatostatin receptor-positive lesions without [18F]-FDG uptake and a grade P5 indicated significant [18F]-FDG-positive/somatostatin receptor-negative disease. The other categories, P2 to P4, were classified as intermediate with a progressive increase in [18F]-FDG uptake (relative to uptake on somatostatin receptor imaging) from P2 to P4 in the target lesion. However, there are no prospective studies published about the NETPET scoring system validity, and there is no correlation between NETPET findings with treatment success and disease-free period.

In this study, we described a case of a patient with a disseminated ACTH-secreting ovarian neuroendocrine tumor. Dual-tracer PET/CT showed a more aggressive phenotype in the primary tumor than in the majority of metastases, even though the immunohistochemical examination demonstrated an equal Ki-67 index (15%) for the primary tumor and the metastases. We suppose that this tumor heterogeneity confirms the hypothesis that the microenvironment of the tumor is important for the expression of different molecular markers.

[68Ga]-DOTA-TATE is considered to be a radiopharmaceutical that predominantly targets the somatostatin receptor 2 subtype. However, in the present case, histological examination revealed only the somatostatin receptor 5 subtype in the tumor cells. This case demonstrated that [68Ga]-DOTA-TATE PET/CT scanning does not always provide precise characteristics of the subtype of somatostatin receptors. Nevertheless, dual-tracer PET/CT allowed the accurate identification of the biological characteristics of a primary tumor and metastases.

The growth control of neuroendocrine tumors can be achieved by somatostatin analogs such as octreotide or lanreotide (20). In our study, treatment with the somatostatin analog alone proved to be ineffective. This is probably due to the fact that the majority of tumor cells overexpressed somatostatin receptor subtype 5. Taking into account the high [18F]-FDG uptake in the primary tumor and metastases, marking tumor aggressiveness, the preferred treatment strategy should be surgical resection and adjuvant chemotherapy.

The follow-up PET/CT imaging was applied for the assessment of treatment efficiency in this study. Nevertheless, laboratory examination of chromogranin A or liquid biopsy may also be helpful in monitoring the response to treatment and in detecting relapses.

## Conclusion

ACTH-producing ovarian neuroendocrine neoplasm is a rare entity. This case had an aggressive clinical course, despite cytoreductive surgical treatment and somatostatin analog therapy. PET/CT imaging with two tracers is a molecular tool that defines a biologic heterogeneity between primary tumor and metastases and yields additional information that may influence the choice of the patient management strategy.

## Data availability statement

The raw data supporting the conclusions of this article will be made available by the authors, without undue reservation.

## Ethics statement

The studies involving human participants were reviewed and approved by the ethics committee of Almazov National Medical Research Center. The patients/participants provided their written informed consent to participate in this study. Written informed consent was obtained from the individual(s) for the publication of any potentially identifiable images or data included in this article.

## Author contributions

DR and UT designed the study. DR and LM collected the data and initially drafted the manuscript. LM, EG and ES reviewed and revised the manuscript. All authors read and approved the submitted version.

## Funding

This work was financially supported by the Ministry of Science and Higher Education of the Russian Federation (Agreement No. 075-15-2022-301).

## Conflict of interest

The authors declare that the research was conducted in the absence of any commercial or financial relationships that could be construed as a potential conflict of interest.

The reviewer MM declared a past collaboration with the authors DR and LM to the handling editor.

## Publisher’s note

All claims expressed in this article are solely those of the authors and do not necessarily represent those of their affiliated organizations, or those of the publisher, the editors and the reviewers. Any product that may be evaluated in this article, or claim that may be made by its manufacturer, is not guaranteed or endorsed by the publisher.

## Abbreviations

ACTH, adrenocorticotropic hormone; AIP, aryl hydrocarbon receptor interacting protein; APC, adenomatous polyposis coli; CT, computed tomography; [68Ga]-DOTA-TATE, [68Ga]-labeled dodecanetetraacetic acid-tyrosine-3-octreotate; NET, neuroendocrine tumor; NSE, neuron-specific enolase; PET-CT, positron emission tomography–computed tomography; 18F-FDG, 18F-fluorodeoxyglucose; SUVmax, maximum standardized uptake value.
